# Retrosplenial Cortex Indexes Stability beyond the Spatial Domain

**DOI:** 10.1523/JNEUROSCI.2602-17.2017

**Published:** 2018-02-07

**Authors:** Stephen D. Auger, Eleanor A. Maguire

**Affiliations:** Wellcome Centre for Human Neuroimaging, Institute of Neurology, University College London, London WC1N 3AR, United Kingdom

**Keywords:** actions, concepts, fMRI, landmarks, retrosplenial, scenes

## Abstract

Retrosplenial cortex (RSC) is highly responsive to landmarks in the environment that remain fixed in a permanent location, and this has been linked with its known involvement in scene and spatial processing. However, it is unclear whether RSC representations of permanence are a purely spatial phenomenon or whether they extend into behavioral and conceptual domains. To test this, during functional MRI scanning, we had people (males and females) read three different types of sentences that described either something permanent or transient. The first two sentence types were imageable, with a focus either on a spatial landmark or on an action. The third type of sentence involved non-imageable abstract concepts. We found that, in addition to being more active for sentences describing landmarks with a permanent location in space, RSC was also significantly engaged by sentences describing stable and consistent behaviors or actions, as long as they were rooted within a concrete imageable setting. RSC was not responsive to abstract concepts, even those that embodied the notion of stability. Similarly, it was not engaged by imageable sentences with transient contents. In contrast, parahippocampal cortex was more engaged by imageable sentences describing landmarks, whereas the hippocampus was active for all imageable sentences. In addition, for imageable sentences describing permanence, there was bidirectional functional coupling between RSC and these medial temporal lobe structures. It appears, therefore, that RSC-mediated permanence representations could be helpful for more than spatially mapping environments and may also provide information about the reliability of events occurring within them.

**SIGNIFICANCE STATEMENT** The retrosplenial cortex (RSC) is known to process information about landmarks in the environment that have a fixed, permanent location. Here we tested whether this permanence response was apparent beyond the spatial domain, which could have implications for understanding the role of the RSC more widely across cognition. We found that the RSC was engaged not only by permanent landmarks but also by stable and consistent actions. It was not responsive to transient landmarks or actions or to abstract concepts, even those that embodied the notion of stability. We conclude that the RSC might do more than help to map spatial environments, by possibly also providing information about the reliability of events occurring within them.

## Introduction

The retrosplenial cortex (RSC) plays a well established role in processing scenes and spatial information (Iaria et al., 2007; Epstein, 2008; Vann et al., 2009; Galati et al., 2010; Auger et al., 2012, 2015; Marchette et al., 2014; Shine et al., 2016; Vedder et al., 2017; Alexander and Nitz, 2017; Jacob et al., 2017; Mao et al., 2017). Indeed, it is commonly included among “scene-selective” brain regions (Dilks et al., 2011; Golomb et al., 2011; Nasr et al., 2011, 2013). It has been suggest that RSC may help localize and orient a scene within wider environmental representations (Park and Chun, 2009; Epstein and Vass, 2014; Hindley et al., 2014). Alternatively, it might make spatial comparisons (Nasr et al., 2013) or translate between allocentric and egocentric representations of space (Byrne et al., 2007; Vann et al., 2009).

It has also been proposed that RSC could, in fact, be concerned with the coding of landmarks that do not move and are permanently located in space, and this may explain its engagement in scene and spatial studies (Auger et al., 2012, 2015, 2017; Auger and Maguire, 2013). To date, however, permanence has only been considered in terms of spatial processing. This leaves important questions regarding the scope and purpose of these representations untested. Given that other brain areas, like the hippocampus, have a clear role in, but also beyond, spatial cognition, in the current study we investigated whether the RSC too might play a more wide-ranging role in indexing permanent, nonlandmark, features and concepts.

Responses analogous to scene selectivity have been demonstrated when people read sentences describing concrete, rather than abstract, situations (Wallentin et al., 2005; Wang et al., 2010). Here, we built on these findings and created three different types of sentences to investigate the scope of RSC permanence representations. The first sentence type was designed to be directly analogous to previous studies that used visual images of landmarks (Auger et al., 2012) and comprised imageable sentences that mentioned either a permanent or transient landmark. The second sentence type, also imageable, focused on permanent or transient actions, rather than landmarks. Finally, there were sentences describing non-imageable abstract concepts but that, nevertheless, varied in terms of their permanence. Participants read these sentences during fMRI scanning. They were unaware of the experimental manipulations and performed an incidental vigilance task.

This sentence-reading paradigm provided scope to examine several issues. First, we could test whether the previous findings of RSC engagement for permanent landmarks were replicated in this sentence-reading task. Next, and of particular interest, we were able to investigate whether RSC was responsive to stimuli where the focus was not on spatial features like landmarks but instead on permanent actions. We could also assess whether the RSC's reach extended even further into the realm of abstract concepts. Two other brain regions often described as being scene selective are parahippocampal cortex (PHC) and the transverse occipital sulcus (TOS; Nasr et al., 2011; Bettencourt and Xu, 2013). Here we could test whether they were engaged by the imageable sentences in general or instead by particular features described in the sentences. Similarly, the hippocampus has been linked with visual imagery and the mental construction of scenes, so we were also interested to know how it would respond to the imageable and non-imageable sentences (Hassabis and Maguire, 2007, 2009; Maguire and Mullally, 2013; Zeidman and Maguire, 2016).

We had a strong hypothesis that RSC would respond to imageable sentences that described permanent landmarks, in line with the previous literature in the visuospatial domain (Auger et al., 2012, 2015, 2017; Auger and Maguire, 2013). Given the dearth of studies examining permanence beyond spatial cognition, it was unclear whether or not RSC would be responsive to sentences describing stable and consistent actions and non-imageable abstract concepts. This study would, therefore, provide novel insights into the parameters within which the RSC operates.

## Materials and Methods

### 

#### Participants

Twenty healthy, right-handed participants with normal or corrected-to-normal vision and who were highly proficient in reading and speaking English (10 females; mean age, 22.2 years; SD, 3.2) took part in the behavioral ratings experiment.

Thirty-two healthy, right-handed participants, none of whom had taken part in the ratings study, participated in the main fMRI experiment (16 females; mean age, 21.6 years; SD, 3.9). All had normal vision and were highly proficient in reading and speaking English.

All participants in both experiments gave written informed consent in line with the policy of the local research ethics committee.

#### Stimuli

**Figure 1.**
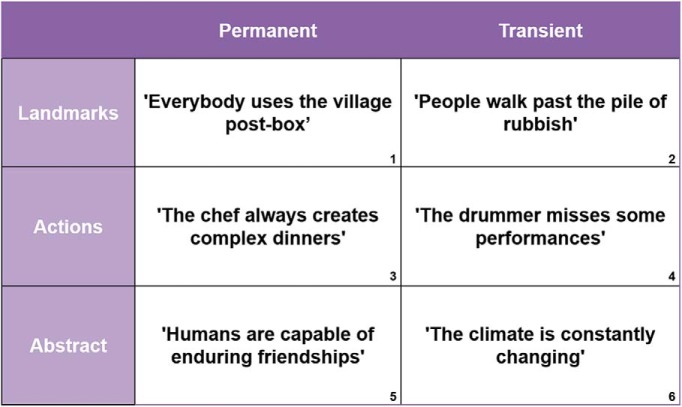
Example sentences from each of the six categories. The numbers are the category labels referred to in the main text.

We first created a set of 344 sentences. There were three different types of sentences, and within each sentence type, there were descriptions that referred to either something permanent or transient, giving a total of six sentence categories (Fig. 1 shows the numbered categories and examples of each). The first sentence type explicitly referred to a spatial feature or landmark (“landmark” condition) that was either permanent or transient; for example, category 1–permanent: “Everybody uses the village post-box,” where the post-box is a landmark that is fixed and does not move; category 2–transient: “People walk past the pile of rubbish,” where the rubbish is transient and will not stay fixed in a location. The second type of sentence (“action” condition) referred to a permanent or transient action; for example, category 3–permanent: “The chef always creates complex dinners,” where the chef reliably performs this action; category 4–transient: “The drummer misses some performances,” where the drummer is not so reliable or stable in terms of this action. The third type of sentence (“abstract” condition) described abstract concepts that were permanent or transient; for example, category 5–permanent: “Humans are capable of enduring friendships,” which implies a reliable and stable concept; category 6–transient: “The climate is constantly changing,” where the climate is not fixed or stable.

This set of sentences was first characterized in a behavioral ratings experiment, and a closely matched set of 300 sentences was selected for use in the fMRI study. For both the ratings and fMRI experiments, sentences were displayed in black, point size 50, Calibri font, in the center of a screen with a gray background.

#### Behavioral ratings experiment

The goal of the ratings experiment was to ensure the six-sentence categories used in the fMRI experiment were matched according to a range of different features. There were two rounds of questioning in which participants rated different features of the sentences. In each round, the 344 sentences were presented in a randomized order one after the other for 4 s, and each was immediately followed by some questions.

The first round of questioning sought to characterize the imageability of the sentences and vividness of any image they evoked. For each sentence, participants were first asked “Does this sentence bring an image of an item or scene into your mind's eye?,” to which they could reply with one of three options: “No image,” an image of a “Single item,” or an image of a “Full scene.” If they indicated that the sentence did bring an image to mind (either of an item or a scene), they were then asked whether this image was “weak” or “strong.”

When designing the sentences, we aimed to ensure that they all referred to ordinary, everyday items/actions and that the descriptions were clear and unambiguous. We tested whether this was the case in the second round of questioning. Participants were again shown the sentences one at a time in a different order. For each one they first rated “Is the thing described in the sentence ordinary?” and chose either “Ordinary” or “Out of the ordinary.” After giving this response, participants were then asked “Does anything else, not mentioned in the sentence, come to mind?”; they indicated either “Yes” or “No,” and if the former, they were then asked what it was that came to mind.

**Figure 2.**
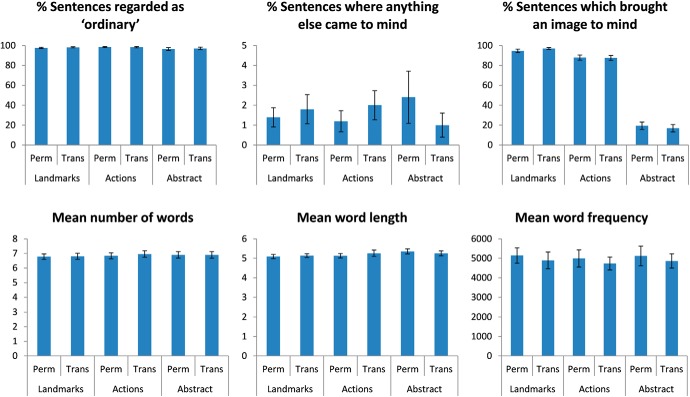
Stimulus matching. The sentences used in the fMRI study were very well matched on a range of features, and, as expected, the sentences in categories 1–4 were significantly more imageable than those in categories 5 and 6. Means +/− 1 SEM.

After collecting this set of ratings, we first excluded any sentences that were consistently considered unusual [where more than five (one-fourth) participants rated it “Out of the ordinary”]. Any sentences in categories 1–4 that more than seven (approximately one-third) participants said it brought no image to mind were also excluded, as these landmark and action sentences were expected to evoke imagery. In contrast, for the abstract sentences (categories 5 and 6) where more than seven participants said it brought an image to mind, those sentences were excluded because we wanted these sentences to be abstract and non-imageable. Finally, we excluded any sentences which more than two people said brought to mind something else that was not mentioned in the sentence. We used these ratings to select an optimized set of 300 sentences that were closely matched across the six categories (50 sentences per category) for the fMRI experiment (Fig. 2).

##### Statistical analyses.

The matching between the six categories was confirmed by a series of two-way ANOVAs with two levels for permanence (permanent and transient) and three levels of sentence type (landmark, action, and abstract). The results were as follows: percentage of sentences regarded as ordinary (main effect of permanence: *F*_(1,294)_ = 1.141, *p* = 0.3; main effect of sentence type: *F*_(2,294)_ = 2.865, *p* = 0.06; interaction: *F*_(2,294)_ = 0.406, *p* = 0.7); number of sentences where anything else came to mind (main effect of permanence: *F*_(1,294)_ = 0.191, *p* = 0.7; main effect of sentence type: *F*_(2,294)_ = 0.191, *p* = 0.8; interaction: *F*_(2,294)_ = 2.479, *p* = 0.09); and percentage of sentences that brought an image into the mind's eye (main effect of permanence: *F*_(1,294)_ = 0.005, *p* = 0.9; main effect of sentence type: *F*_(2,294)_ = 1155.068, *p* < 0.001, partial Eta-squared effect size = 0.887; interaction: *F*_(2,294)_ = 0.918, *p* = 0.4). Note that a significant difference between sentences in categories 1–4 versus categories 5 and 6 was expected as the sentences were specifically designed to have different imageability. This difference was also reflected in an ANOVA demonstrating a greater tendency for the sentences in categories 1–4 to bring a “Full scene” to mind than categories 5 and 6 (main effect sentence type: *F*_(1,296)_ = 246.174, *p* < 0.0001, partial Eta-squared effect size = 0.454; main effect of permanence: *F*_(1,296)_ = 0.256, *p* = 0.6; interaction: *F*_(1,296)_ = 0.008, *p* = 0.9). On occasions, we therefore refer to sentences in categories 1–4 as imageable and those in categories 5 and 6 as non-imageable.

The sentence categories were further matched for the properties of the words they contained, namely the number and length of words: mean number of words (main effect of permanence: *F*_(1,294)_ = 0.071, *p* = 0.8; main effect of sentence type: *F*_(2,294)_ = 0.176, *p* = 0.8; interaction: *F*_(2,294)_ = 0.045, *p* = 1.0) and mean word length (main effect of permanence: *F*_(1,294)_ = 0.070, *p* = 0.8; main effect of sentence type: *F*_(2,294)_ = 1.180, *p* = 0.3; interaction: *F*_(2,294)_ = 0.409, *p* = 0.7).

We also matched the sentences for word frequency to ensure that the sentences did not differ in the amount of rare or uncommon words they contained. For this, we used a frequency list generated from the 100 million-word British National Corpus (Kilgarriff, 1997). Specifically, the six categories were carefully matched for the mean word frequency of their low-frequency words (those with a frequency <0.025%; main effect of permanence: *F*_(1,294)_ = 0.576, *p* = 0.4; main effect of sentence type: *F*_(2,294)_ = 0.081, *p* = 0.9; interaction: *F*_(2,294)_ = 0.000, *p* = 1.0).

This provided us with a set of thoroughly characterized sentences, which were rigorously matched.

#### Stimuli and task for the fMRI experiment

For the fMRI experiment, in addition to the optimized set of 300 sentences from the ratings study, we included 50 additional nonsensical sentences to serve as the basis of an incidental vigilance task that would be performed during scanning, e.g., “Looking through murky with market planning.” The nonsense sentences were specifically designed so that their first few words could potentially form a meaningful sentence (e.g., “Looking through murky”) so that it was not immediately obvious if a sentence was nonsense. This ensured that participants had to read sentences in full to establish their meaning. In this way, we could be confident that everyone was reading all the sentences in their entirety. The 50 nonsense sentences were also completely matched to sentences in the six main categories for the length, number, and frequency of their words (mean word length: *F*_(6,343)_ = 0.826, *p* = 0.6; mean number of words: *F*_(6,343)_ = 0.410, *p* = 0.9; mean word frequency: *F*_(6,343)_ = 0.571, *p* = 0.8).

While undergoing fMRI scanning, participants were presented with the 350 sentences and performed a simple vigilance task. They were instructed to read each sentence carefully and press a button if the sentence they were reading was “complete and utter nonsense.” This ensured that they paid attention to the sentences and their meaning, but without drawing attention to any particular sentence feature. No mention was made about any of the key differences between the sentences, and there were no instructions that they should try to picture what was being described. This ensured that participants were completely naive about the key manipulations of interest, which was crucial to allow an unbiased assessment of neural responses evoked by the different sentence categories.

The 350 sentences were presented one at a time for 4 s each in a pseudorandomized order; this was achieved by randomizing stimulus order until there was an even distribution of the six categories and nonsense sentences across the whole scanning period. There was a 2–4 s jittered interval separating the sentences during which a black fixation cross was shown in the center of a gray background. Participants pressed a button with their right index finger if they thought the sentence they were reading was nonsense and were instructed to do nothing for sentences they thought were sensible. Pressing the button caused the trial to immediately end and move on to the next intertrial fixation period. All nonsense sentences and any extra trials on which subjects pressed the button were removed from the fMRI analysis. The 350 trials were split into three scanning runs of ∼13.5 min each.

Immediately after scanning was completed, participants were debriefed. The aim of this debriefing session was to ascertain whether or not they had become aware of the key differences between sentences while performing the incidental vigilance task. Participants were first asked: “Did you notice anything in particular about the sentences?” If they did not articulate any of the differences, participants were then asked more specifically: “Did you notice any definite pattern in what the sentences described or were they just random?” Finally, they were presented with a list of 12 different possible ways in which the sentences might have varied and were instructed to identify one of the options they thought was correct. Eleven of the options were incorrect foils (e.g., “the type of font the sentences were written in”), and there was only one correct option (“described either permanent or transient things”). Participants then had to indicate how confident they were in their selection (“Guessing,” “Not confident,” “Fairly confident,” “Very confident”).

#### Scanning parameters and preprocessing

T2*-weighted echo planar images (EPI) with BOLD contrast were acquired on a 3T whole-body MRI scanner (Magnetom TIM Trio; Siemens Healthcare). We used a 32-channel head receive coil and the standard Radio Frequency (RF) transmit body coil. Scanning parameters were optimized for the hippocampus and surrounding tissue while still achieving whole-brain coverage: 48 oblique axial slices angled at −45° from the axial to coronal plane [as defined by Weiskopf et al. (2006)]; 2.5 mm thickness (with interslice distance factor of 20%); repetition time (TR), 3.36 s (slice TR, 70 ms); echo time (TE), 30 ms; echo spacing, 500 μs; matrix size, 64 × 74; 13% oversampling in the phase encoding (PE) direction; excitation flip angle, 90°; in-plane resolution, 3 × 3 mm; field of view, 192 × 192 mm PE in the anteroposterior direction. For reduction of signal loss in the hippocampal region, slices were angulated and a *z*-shim gradient moment of +0.6 mT/m*ms was applied (Weiskopf et al., 2006). The first six “dummy” volumes from each scanning run were discarded to allow for T1 equilibration effects. Field maps were acquired with a standard manufacturer's double-echo gradient echo field map sequence (short TE, 10 ms; long TE, 12.46 ms; 64 axial slices with 2 mm thickness and 1 mm gap yielding whole-brain coverage; in-plane resolution, 3 × 3 mm). A 3D modified driven equilibrium Fourier transform (MDEFT) T1-weighted structural scan (Deichmann et al., 2004) was acquired for each participant with 1 mm isotropic resolution. fMRI data were analyzed using SPM8 (www.fil.ion.ucl.ac.uk/spm; RRID:SCR_007037). Images were bias corrected, realigned and unwarped (using the field maps), normalized to a standard EPI template in MNI space with a resampled voxel size of 3 × 3 × 3 mm, and smoothed using an 8 mm FWHM Gaussian kernel.

#### fMRI: first- and second-level statistics

After preprocessing, we performed a series of whole-brain univariate fMRI analyses using a general linear model. Each trial was modeled as the full 4 s that a sentence was on display, and we applied the default SPM high-pass filter cutoff of 128 s with no global normalization.

Each of the univariate fMRI contrasts described in the sections below were run using the same general linear model, with one regressor of interest for each of the six sentence types. A separate regressor was included for the nonsense sentences and any trial on which the button was pressed (i.e., those that a participant thought were nonsense). This regressor and the individual movement regressors were treated as covariates of no interest. Regressors of interest were convolved with the canonical hemodynamic response function. Subject-specific parameter estimates pertaining to each contrast (betas) were calculated for each voxel. Second-level random effects analyses were run on these parameter estimates (collapsing across scanning runs) using one-sample *t* tests. We report any fMRI activations that survived a whole-brain family-wise error (FWE)-corrected threshold of *p* < 0.05 unless otherwise stated. Given our strong hypotheses regarding engagement of the RSC in relation to permanence, we report any increased activity in this region at a whole-brain uncorrected threshold of *p* < 0.001 for contrasts pertaining to permanence.

#### fMRI: interaction between sentence type and permanence

In the first instance, we considered fMRI responses in relation to the interaction between whether sentences were imageable (categories 1–4) or not (categories 5 and 6) and their permanence. We then performed a second interaction analysis, this time only considering imageable sentences, to look for responses that were sensitive to permanence and whether sentences described a landmark (categories 1 and 2) or an action (categories 3 and 4).

#### fMRI: differences between sentence types

We examined responses related to the type of sentence, independent of whether what was being described was permanent or transient. To do this, we compared imageable sentences (categories 1–4) with non-imageable sentences (categories 5 and 6). Within the imageable categories, we also contrasted fMRI responses to landmark sentences (categories 1 and 2) with action sentences (categories 3 and 4).

#### fMRI: differences in permanence

We then examined responses associated with permanence. To do this, we first performed separate analyses of permanent and transient sentences. We contrasted permanent imageable sentences (categories 1 and 3) with permanent non-imageable sentences (category 5) and permanent landmark sentences (category 1) with permanent action sentences (category 3). We did the same for the transient sentences. Next, we directly compared permanent with transient imageable sentences (categories 1 and 3 vs categories 2 and 4). In addition, we separately contrasted permanent and transient landmark (category 1 vs category 2) and action (category 3 vs category 4) sentences. For all the permanent versus transient sentences contrasts described, we also performed corresponding analyses to look for any regions that might be more active for transient sentences.

#### Connectivity analyses

For the regions shown to be engaged by imageable sentences, we then examined how they interacted with other brain areas in relation to the permanence of what was being described. Each of the activation clusters identified in the univariate analyses (at a threshold of *p* < 0.001 for RSC and *p* < 0.05 FWE corrected for the whole brain) were used as seed regions in generalized psychophysiological interaction (gPPI) analyses (McLaren et al., 2012). Specifically, we looked for any brain areas that had increased functional coupling with the seed regions on permanent compared with transient trials. The precise contrast used for each seed region corresponded to the univariate contrast from which they were derived. The gPPI analyses were performed using the “Generalized Form of Context-Dependent Psychophysiological Interactions” SPM toolbox (McLaren et al., 2012), and we report any significant results at a whole-brain uncorrected threshold of *p* < 0.001 for the RSC (given our specific prior hypotheses regarding permanence processing in this region) and *p* < 0.05 FWE corrected for the rest of the brain.

Any significant interactions between regions identified in the gPPI analyses were further examined using dynamic causal modeling (DCM; Friston et al., 2003). DCM allows the comparison of different models of task-dependent effective connectivity between prespecified brain areas. We specifically investigated the permanence-based causal influence between brain regions already shown to have permanence-related interactions with one another (from the gPPI analyses). We used stochastic DCM (Daunizeau et al., 2012), which accounts for endogenous fluctuations in brain activity. This is of particular relevance here as our participants were not directly viewing what was described in the sentences, so much of the network's activity will have been driven by endogenous brain processes (rather than purely external experimental manipulations).

The design matrix used for the DCM analysis contained two main regressors: one for all imageable sentences (categories 1–4) and a second for just permanent imageable sentences (categories 1 and 3). The first was to be used for the models' input (the DCM C matrix) and the second for modulatory connections (B matrix). DCM10 was used for the analysis, and we assumed there to be reciprocal endogenous connections between the regions as well as self-connections (A matrix). All plausible models of interaction between the regions were compared. These differed in the connections that were modulated by permanence (B matrix) and the region that received the system's driving input (C matrix; see Fig. 5*B* for exact model architectures). Each of the models' predicted hemodynamic responses were fitted to the actual fMRI data in each participant and compared using a random-effects Bayesian model comparison to establish the most likely “winning” model (Stephan et al., 2009).

## Results

### Behavioral

Behavioral responses made by participants while they were performing the vigilance task inside the scanner indicated that they successfully maintained attention. The mean error rate (missed or inappropriate “nonsense” responses) was very low throughout for all participants (mean error rate, 3.1%; SD, 2.9).

After scanning, participants were asked whether they noticed any patterns or differences in what the sentences described. None of the 32 participants made reference to being aware of any difference in whether sentences described something permanent or transient. When presented with a list of 12 options for the way in which the sentences could have varied, 7 of the 32 participants correctly identified that the sentences described something either permanent or transient. All of those seven participants indicated that they only considered this after seeing it on this list of options in the debriefing, and even then they were not particularly confident in their choice: four stated they were “Not confident,” three were “Fairly confident,” and none were “Very confident.” Thus, during fMRI scanning, no participant seemed to have been consciously aware of the crucial distinction between permanent and transient sentences, and even when aided, only three could pinpoint the distinction with any confidence. Therefore, any neural responses related to the key features of the sentences are likely to be from automatic, implicit processing.

### fMRI: interaction between sentence type and permanence

From the data generated in the behavioral ratings study, we knew that the sentences in categories 1–4 were imageable whereas those in categories 5 and 6 were non-imageable.

We first performed an analysis to examine fMRI responses in relation to the interaction between a sentence's permanence and whether it was imageable or not. Significant activations in bilateral RSC (right: 18, −43, 10; *Z* = 5.20; left: −15, −52, 13; *Z* = 4.57) were evident. No other brain region showed increased activity at a FWE-corrected threshold of *p* < 0.05. A second interaction analysis considered responses in relation to whether an imageable sentence described a landmark or an action and the permanence of what was being described. No regions were responsive to this interaction, even at a reduced whole-brain uncorrected threshold of *p* < 0.001.

These results indicate that RSC may be sensitive to the interaction between whether or not a sentence is imageable and its permanent content, but not whether an imageable sentence refers to either a landmark or action. To formally assess these findings further, we then directly interrogated responses in relation to sentence type and permanence separately.

### fMRI: differences between sentence types

**Figure 3.**
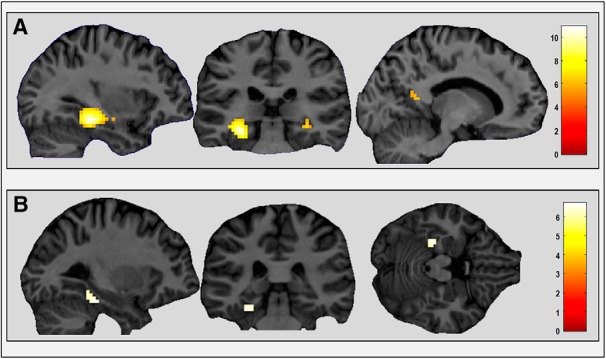
Brain regions responsive to sentence type. ***A***, PHC, extending into hippocampus (left and middle) and a small cluster in RSC (right) showed greater activity for imageable sentences (categories 1–4) than non-imageable sentences (categories 5 and 6). ***B***, Left PHC showed greater activity for imageable sentences describing a landmark (categories 1 and 2) than those describing an action (categories 3 and 4). Activations are shown on views from a single representative participant's structural MRI brain scan, displayed at a whole-brain FWE-corrected threshold of *p* < 0.05. The color bar indicates the *Z*-scores associated with each voxel.

Comparing responses to imageable sentences (categories 1–4) with non-imageable sentences (categories 5 and 6) revealed increased activity in parts of the cortex traditionally labeled as being “scene selective,” the PHC bilaterally (left: −30, −31, −14; *Z* = 10.96; right: 33, −28, −11; *Z* = 6.59) and left RSC (−9, −52, 10; *Z* = 5.93; Fig. 3*A*). The bilateral PHC clusters also extended into the hippocampus. The TOS did not show increased engagement for imageable sentences. The reverse contrast (categories 5 and 6 vs categories 1–4) revealed no significant activation at a FWE-corrected threshold of *p* < 0.05, but at a whole-brain uncorrected threshold of *p* < 0.001, clusters in medial prefrontal (9, 68, 10; *Z* = 5.54), superior posterior parietal (−18, −52, 46; *Z* = 5.03), and anterior cingulate (−3, 11, −8; *Z* = 4.78) cortices were more active for abstract than imageable sentences.

Within the imageable sentences, if the focus was on a landmark (categories 1 and 2), then the left PHC (−27, −34, −20; *Z* = 6.69; Fig. 3*B*) had increased activity compared with imageable sentences describing actions (categories 3 and 4). This indicates that PHC may, in particular, process space-related features.

Thus, RSC, PHC, and hippocampus were engaged when people read imageable sentences. This response was especially apparent in PHC and was present when the sentence had a particular focus on a spatial landmark rather than an action. So whereas PHC processing appears to be concerned with spatial features, RSC and hippocampus are perhaps engaged by other factors.

### fMRI: differences in permanence

Having established strong responses to imageable sentences in traditional scene-selective cortex, we probed these representations further, taking into account the permanence of what the sentences described. For the contrast that demonstrated increased activity within RSC, PHC, and hippocampus (imageable vs non-imageable sentences), we now separated out permanent and transient sentences. Transient imageable sentences (categories 2 and 4) produced significantly greater activity in PHC (left: −30, −31, −11; *Z* = 12.52; right: 36, −34, −11; *Z* = 7.07) and left posterolateral parietal cortex (−45, −76, 34; *Z* = 10.94) than transient non-imageable sentences (category 6). Similar to the responses observed for all imageable sentences combined (both permanent and transient together), the clusters in PHC extended into parts of the hippocampus. However, unlike the combined permanent and transient imageable sentences, transient imageable sentences did not engage RSC more than transient non-imageable sentences, even at a more liberal whole-brain uncorrected statistical threshold of *p* < 0.005. Therefore, if a sentence described something transient, RSC lost responsivity to sentences, even those that were imageable.

For sentences describing something permanent, a contrast of imageable sentences (categories 1 and 3) compared with non-imageable sentences (category 5) revealed increased activity within bilateral PHC/hippocampus (left: −33, −37, −8; Z = 10.77; right: 33, −37, −5; Z = 7.12), bilateral RSC (left: −9, −55, 16; *Z* = 8.95; right: 9, −52, 19; *Z* = 6.61), as well as bilateral posterolateral parietal cortex (left: −42, −76, 34; *Z* = 9.04; right: 45, −61, 28; *Z* = 7.40) and right inferior–lateral parietal cortex (54, −7, 10; *Z* = 8.85).

**Figure 4.**
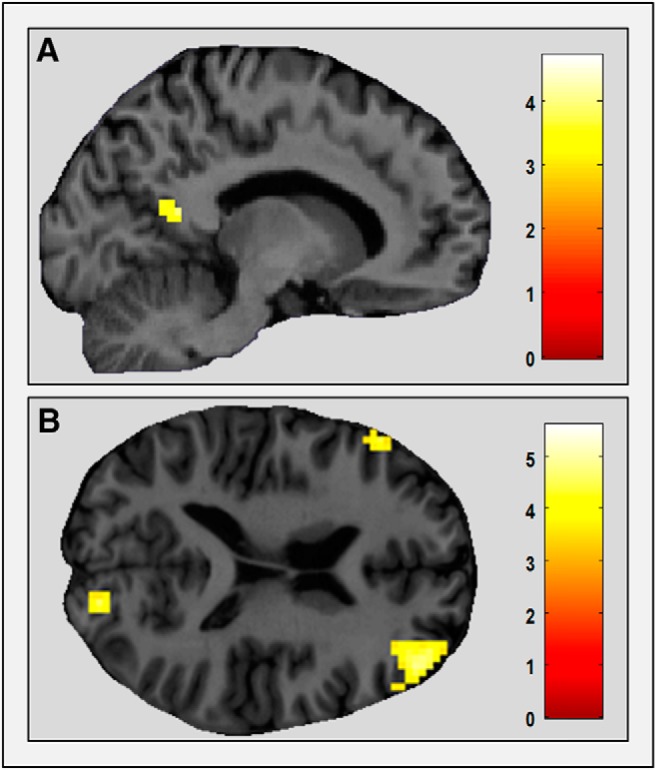
Brain regions responsive to permanence. ***A***, RSC was more engaged by permanent than transient imageable sentences (categories 1 and 3 vs categories 2 and 4). ***B***, DLPFC and posterior occipital cortex were more active for permanent than transient non-imageable abstract sentences. Activations are shown on sagittal and axial views, respectively, of a single representative participant's structural MRI brain scan, displayed at a whole-brain threshold of *p* < 0.001 (uncorrected, for display purposes). The color bar indicates the *Z*-scores associated with each voxel.

Whereas PHC and hippocampus were responsive to all imageable sentences, either permanent or transient, RSC was only engaged when the landmark or action described in a sentence was permanent. We then contrasted fMRI responses to permanent and transient sentences directly. There was significantly greater activity in bilateral RSC (left: −12, −52, 13; *Z* = 3.92; right: 18, −46, 10; *Z* = 3.77), but not any other brain region, for permanent compared with transient imageable sentences (categories 1 and 3 vs categories 2 and 4; Fig. 4*A*).

We also directly compared permanent and transient sentences separately for landmark and action sentences. Increased activity in RSC, but no other brain region, was still evident for permanent compared with transient landmarks (category 1 vs category 2: 21, −43, 4; *Z* = 3.46) and permanent compared with transient actions (category 3 vs category 4: −9, −52, 16; *Z* = 3.46). For permanent versus transient non-imageable abstract sentences (category 5 vs category 6), there was no difference in RSC activity, even at the reduced whole-brain uncorrected threshold of *p* < 0.005. At a whole-brain uncorrected threshold of *p* < 0.001, bilateral dorsolateral prefrontal cortex (DLPFC) was more active for permanent than transient abstract sentences (right: 42, 53, 7; *Z* = 5.37; left: −51, 44, 4; *Z* = 4.95; Fig. 4*B*) as well as posterior parts of the occipital lobes (right: 15, −85, 10; *Z* = 5.58; left: −9, −88, 4; *Z* = 4.93) and the cerebellum (−36, −58, −47; *Z* = 4.80).

We also compared landmark and action permanent and transient sentences separately (categories 1 vs category 3; category 2 vs category 4). Here, when the permanence of sentences being compared was the same, PHC was the only region more active for landmark than action sentences for both permanent (left: −27, −31, −20; *Z* = 4.83; right: 30, −34, −17; *Z* = 4.02) and transient (−30, −34, −20; *Z* = 5.63) sentences (at a whole-brain uncorrected threshold of *p* < 0.001), with no evidence of RSC engagement even at the more liberal uncorrected whole-brain threshold of *p* < 0.005.

We also performed similar comparisons with all those mentioned above, but looking instead for brain areas more engaged by transient than permanent sentences. None of these comparisons revealed any significant activation in any brain region.

RSC was, therefore, consistently more engaged by permanent than transient sentences but only if the sentences described something that was imageable. This was the case when comparing both permanent and transient landmarks and also actions. Indeed, RSC lost all sensitivity to imageable sentences if what was being described was transient. No similar sensitivity to permanence was evident in PHC or hippocampus; both were more responsive to sentences that were imageable regardless of permanence. For non-imageable abstract sentences, albeit at a more liberal threshold, the DLPFC, but not RSC, was more active if they described something permanent.

### Connectivity between brain regions associated with permanence

We next performed a gPPI analysis using regions identified in the whole-brain univariate contrasts as seeds. Specifically, we assessed how the interactions with other brain regions might differ for areas shown to be responsive to imageable sentences (bilateral PHC/hippocampus and left RSC) and sentences describing landmarks (left PHC) depending on permanence.

**Figure 5.**
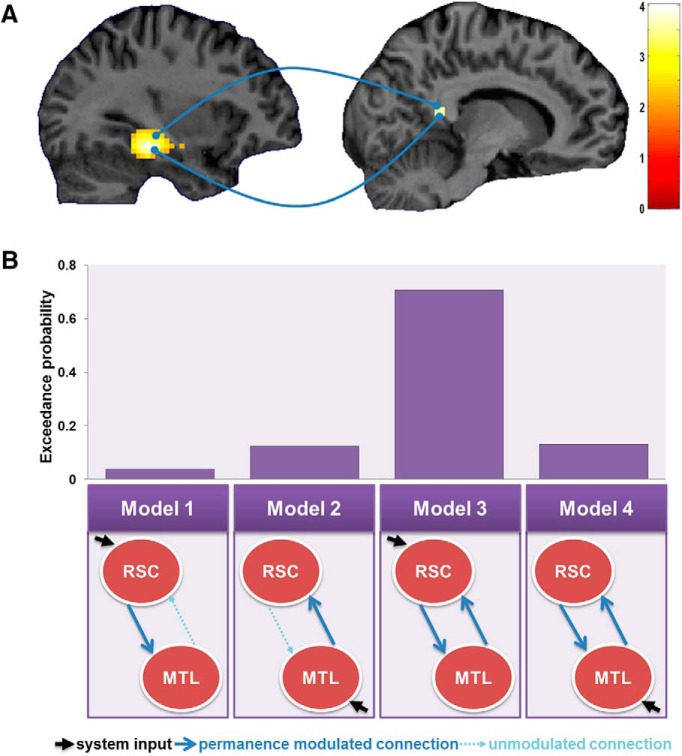
Connectivity analyses. ***A***, Results of the gPPI analysis. RSC (right) had greater functional coupling with medial temporal lobe regions (PHC and hippocampus; left) for permanent imageable sentences. Activations are displayed on sagittal views of a single representative participant's structural MRI brain scan. The RSC gPPI activation is displayed at a whole-brain uncorrected threshold of *p* < 0.001, and the color bar indicates *Z*-scores associated with each voxel. The medial temporal lobe seed region is taken from the whole-brain univariate contrast of imageable sentences (categories 1–4) versus non-imageable sentences (categories 5 and 6) displayed at the whole-brain FWE-corrected threshold of *p* < 0.05, which is also displayed in Figure 3*A*. ***B***, The dynamics of permanence-related interactions. Four models of RSC–MTL interactions were compared in a DCM analysis (bottom) with their corresponding exceedance probabilities (top). Model 3 was the winning model, suggesting RSC and MTL mutually modulated each other's activity when an imageable sentence involved something permanent, with input to the system coming through RSC.

The bilateral clusters in PHC (extending into hippocampus) that were more engaged when people read imageable sentences than non-imageable sentences also displayed greater functional coupling with RSC if the sentences described something permanent (−12, −52, 7; *Z* = 3.98; Fig. 5*A*). For the part of left PHC that was more engaged by landmarks than action sentences, if that sentence described a permanent rather than a transient landmark, then it also had increased functional coupling with RSC (15, −46, 10; *Z* = 4.01). Neither of the gPPI analyses using RSC seed regions showed any significant changes in functional coupling for permanent versus transient sentences. This perhaps reflects a lack of any brain regions, other than the RSC itself, that were responsive to permanence. We also performed equivalent whole-brain gPPI contrasts to look for greater functional coupling for transient compared with permanent sentences. No significant interactions were present for any of the seed regions. The changes in functional coupling were specific to permanent trials.

Thus, across separate measures, the parts of the medial temporal lobe (MTL) that were responsive to imageable sentences and also imageable sentences describing landmarks displayed increased functional connectivity with RSC when what was being described was permanent. When a sentence was permanent, therefore, RSC was not only more engaged but also increased its interaction with other MTL regions.

Having established the functional coupling between RSC and MTL regions specifically for imageable sentences describing something permanent, we used stochastic DCM to assess the nature of this interaction. The specific regions used in the DCM analysis were the bilateral parts of the MTL (consisting of bilateral PHC, extending into hippocampus), which were more engaged by imageable than non-imageable sentences, and the RSC, which was more active for permanent than transient imageable sentences. Four simple, biologically plausible models of the interaction between these two regions were compared. Motivated by the mass-univariate and gPPI analyses, the DCM analysis considered only imageable trials (categories 1–4) and investigated how interactions between RSC and MTL were modulated when an imageable sentence described something permanent (categories 1 and 3). The structures of the four models were as follows (Fig. 5*B*): Model 1 had RSC as the input region and RSC then driving responses in MTL for permanent imageable sentences; Model 2 was the same but in the opposite direction, with input coming through MTL and MTL then driving RSC permanence responses; Models 3 and 4 had bidirectional modulatory connections, but with the driving input to the system coming through RSC and PHC, respectively. The winning model was Model 3 (Model 3 exceedance probability was 0.71; Models 1, 2, and 4 exceedance probabilities were all <0.15), and this accounted for an average of 89.5% (SD, 4.3) of the variance in the participants' fMRI data. This indicates that for imageable sentences describing something permanent, RSC and MTL were modulating each other's responses, but the input to this system came through RSC.

## Discussion

The primary aim of this study was to examine whether the RSC response to permanent, fixed landmarks extended to other stable contexts; in this case, reliably performed actions and abstract concepts. We found that in addition to being more active for sentences that described landmarks that maintained a permanent location in space, RSC was also significantly engaged by sentences describing permanent behaviors or actions, as long as they were rooted within a concrete imageable setting. RSC was not responsive to abstract concepts, even those that embodied the notion of stability. Similarly, it was not engaged by imageable sentences with transient contents. It appears, therefore, that RSC-mediated permanence representations could be helpful for more than spatially mapping environments and might potentially provide information about the reliability of events occurring within them.

On first impressions, the responses in RSC could appear to reflect scene selectivity (Dilks et al., 2011; Golomb et al., 2011; Nasr et al., 2011, 2013), given that in the behavioral rating study the imageable sentences were typically rated as evoking images of full scenes rather than single objects. This is also consistent with previous demonstrations of RSC processing concrete rather than abstract sentences (Wallentin et al., 2005; Wang et al., 2010). Indeed, there was greater activity in RSC when comparing fMRI responses for all imageable sentences compared with non-imageable abstract sentences. However, this masked a more nuanced reality. For sentences that described something transient, there was no longer any difference between activity elicited by imageable and non-imageable sentences in RSC. The apparent scene selectivity was, in fact, only manifest within a permanent context. This suggests that the role of RSC in scene processing may reflect the processing of permanent features (Auger and Maguire, 2013). RSC processing of scenes has previously been proposed to center around translating between allocentric and egocentric representations of space (Byrne et al., 2007; Vann et al., 2009). However, this cannot account for the difference we observed related to imageable sentences involving permanent actions. Therefore, although RSC might play some role when translating between spatial reference frames, this may not be the only function of this region.

Instead, the present study provides further evidence that RSC is primarily involved in processing permanent, reliable features encountered in our surroundings. Our previous experiments have consistently demonstrated that RSC processes landmarks that remain fixed in a single, permanent location, in real-life (Auger et al., 2012; Auger and Maguire, 2013) and virtual reality (Auger et al., 2015, 2017) environments. Here, RSC was, once again, more engaged by permanent than transient items even when they were simply described in a sentence. This sensitivity to permanent landmarks did not require them to be used for any sort of complex spatial manipulation, localizing or orientating (Nasr et al., 2013; Epstein and Vass, 2014); just a mere reference to them sufficed.

This experiment also revealed that RSC permanence representations appear to extend beyond the purely spatial domain. RSC was not only more engaged by permanent than transient items, but also for sentences describing a permanent, regular action. The responsivity to permanence did not, however, extend to more abstract concepts, so it seems that RSC involvement requires some sort of grounding within concrete, tangible settings. It remains to be seen just how extensive RSC representations of permanent elements are within imageable or scene-type settings. It is possible that they constitute only a minor byproduct of a neural system whose primary function is to identify reliable landmarks for mapping space. However, if RSC permanence processing is indeed more generalized, this would have intriguing implications about the nature and scope of the region's overall contribution to cognition. RSC processing might, for example, help inform more wide-ranging models, such as predictive coding, concerning the perception of surprising or unpredictable events to help optimize representations of environments and the behavior happening within them (Friston, 2010).

The PHC was more active for imageable compared with non-imageable sentences, which could reflect its scene selectivity, given the imageable sentences typically evoked scene imagery. However, here also the story was not so simple. PHC showed a preference, not for permanence but for sentences that described spatial features (in this case, landmarks). This resonates with previous work that linked PHC to the processing of local space around environmental features (Kravitz et al., 2011; Mullally and Maguire, 2011).

Interestingly, another brain region that is frequently considered scene selective is the TOS, yet here this region was not recruited. This might be attributable to the stimuli that we used. Investigations of scene processing in TOS have typically used images of scenes (Dilks et al., 2011; Golomb et al., 2011; Nasr et al., 2011; Bettencourt and Xu, 2013), whereas in the current study, the only visual inputs were words. TOS might, therefore, be involved in lower-level processing of a scene's visual features. This would also be consistent with its close proximity to posterior visual regions. Any imagery that might be generated when reading the sentences could only have been created from purely endogenous representations.

This constructive process might perhaps account for the engagement of the hippocampus for imageable more than non-imageable sentences. It has been proposed that a function of the hippocampus may be to construct representations of scenes in the imagination, and this may help account for its involvement in autobiographical memory, spatial navigation, and imagining the future (Addis et al., 2007; Hassabis and Maguire, 2007, 2009; Schacter et al., 2012; Maguire and Mullally, 2013; Zeidman and Maguire, 2016). If hippocampal engagement truly reflected the construction of scene imagery, then it follows that its responses should not differ depending on whether an imageable sentence described something permanent or transient or described landmarks or actions. This was indeed the case.

The specific features of imageable sentences to which the different brain regions were responsive were also linked to the way in which they interacted with one another. A gPPI analysis showed that the active parts of the MTL (bilateral PHC extending into hippocampus) interacted with RSC if sentences described something permanent. A DCM analysis indicated that this interaction was bidirectional, with the RSC and MTL mutually influencing one another's activity for permanent imageable sentences, but the input driving the system came through RSC.

This interaction could reflect a system whereby dependable cues are first identified within RSC and then integrated into more detailed internal models in the MTL. The ongoing exchange of information between the brain areas could then reflect updating and evaluation of existing neural representations, ensuring their long-term reliability by adapting to what is currently being perceived. In previous experiments, a similar interaction between the RSC and hippocampus was also demonstrated in a purely spatial context (Auger et al., 2015). In this instance, RSC was able to rapidly acquire new permanence representations for previously novel spatial cues. RSC–hippocampal interactions were then linked to the processing of detailed knowledge about the specific locations of permanent landmarks within an environment. The present study indicates that the same network could perhaps contribute to representations of more than just spatial relationships (in this case, also stable actions).

The RSC and MTL were not engaged when people read the abstract sentences, even those that conveyed a sense of permanence. Instead, prefrontal regions responded to the abstract concepts. The medial prefrontal cortex was more active for abstract non-imageable compared with imageable sentences, whereas DLPFC was more active when people read abstract sentences that described something permanent as opposed to transient. This was the only permanent–transient contrast that did not engage RSC, which perhaps reflects the absence of a concrete spatial setting. It is interesting that the DLPFC, which shares dense reciprocal connectivity with RSC (Kobayashi and Amaral, 2003), would instead be more active. Of note, this dense connectivity is not evident in rodents, only in primates (Van Groen and Wyss, 1990, 1992, 2003).

It is tempting to speculate that this could perhaps reflect a system that has evolved in primates to perform more abstract conceptual thinking but that still bears some association to lower-level processing of similar themes in the spatial domain. Thus, whereas RSC processes permanence that is physically embodied (landmarks, actions), the DLPFC might assume such processing for higher-level cognition (Powell et al., 2017). However, further work is clearly required to establish the validity of this conjecture. It may also be possible in the future to leverage ultra-high-resolution fMRI scanning to examine whether separable subregions within the human RSC respond to permanent landmarks and actions.

In summary, this study builds on a previous body of work that indicated that RSC specifically processes permanent, stable environmental landmarks. By having participants read simple sentences while undergoing fMRI scanning, we were able to expand on these findings and establish the generalizability of RSC engagement to also include actions occurring within imageable settings. The responses and interactions between brain regions that we observed occurred with participants performing a completely incidental task. This suggests that the neural processes under consideration are fundamental and automatic. That the RSC might contribute to cognition in a more wide-ranging manner than previously thought offers intriguing new avenues for future inquiry.
